# Recurrent Wernicke's Encephalopathy in a 16-Year-Old Girl with Atypical Clinical and Radiological Features

**DOI:** 10.1155/2014/582482

**Published:** 2014-02-10

**Authors:** S. Lamdhade, A. Almulla, R. Alroughani

**Affiliations:** ^1^Division of Neurology, Department of Medicine, Amiri Hospital, Arabian Gulf Street, Kuwait City, Kuwait; ^2^Neurology Clinic, Dasman Diabetes Institute, Dasman, Kuwait

## Abstract

*Background*. Wernicke's Encephalopathy (WE) is a clinical diagnosis with serious neurological consequences. Its occurrence is underestimated in nonalcoholics and is uncommon in adolescents. We aim to draw the attention to a rare case, which had additional clinical and radiological features. *Case*. A 16-year-old girl presented with three-week history of vomiting secondary to intestinal obstruction. She developed diplopia soon after hospitalization. Neurological evaluation revealed restriction of bilateral lateral recti with horizontal nystagmus, and bilateral limb dysmetria. Brain MRI was normal. She had prompt improvement to thiamine. Four months later, she presented with headache, bilateral severe deafness, and tinnitus. Clinically, she had severe sensorineural hearing loss, bilateral lateral recti paresis, and gait ataxia. CT head showed bilateral caudate nucleus hypodensities. MRI brain revealed gadolinium enhancement of mamillary bodies and vermis. She had significant improvement after IV thiamine. Headache completely resolved while the ocular movements, hearing, and tinnitus improved partially in 72 hours. *Conclusions*. Recurrent WE in adolescence is uncommon. Headache, tinnitus, and deafness are rare clinical features. Although MRI study shows typical features of WE, the presence of bilateral caudate nuclei hypodensities on CT scan is uncommon. Prompt treatment with thiamine is warranted in suspected cases to prevent permanent neurological sequelae.

## 1. Introduction

The clinical description of Wernicke's encephalopathy (WE) was first given by Karl Wernicke in 1881, who described 3 cases; one of them was interestingly nonalcoholic. As more cases were described, its relation to dietary deficiency factors became more obvious.

Nutritional deficiency due to alcoholism accounts for 90% cases of WE. Some misconceptions still persist as it is a rare condition mostly seen in alcoholics. In the last two decades, nonalcoholic WE had been described with various etiological conditions secondary to wrong feeding formula in infants, acute pancreatitis, anorexia nervosa, Crohn's disease, thyrotoxicosis, long term use of TPN following bariatric or major surgeries, bone marrow transplant, AIDS, and chronic kidney disease with hemo- and peritoneal dialysis [1, 2]. In a recent review of 625 cases of non-alcoholic WE, neoplastic disease accounted for 18.1%, followed by GI surgery, hyperemesis gravidarum and fasting/malnutrition in 16.8%, 12.2%, and 10.2% of the cases [3]. Although recurrent WE was described in alcoholics [4], there were only few reported cases of recurrent nonalcoholic WE [5].

Typically, eye signs (ophthalmoplegia), ataxia, and mental status changes represent the cardinal features of WE. However many atypical presenting features had been recently described in the literature, especially in nonalcoholic patients. We describe a case of recurrent nonalcoholic WE with atypical clinical features of severe bilateral hearing impairment and atypical radiological MRI findings of mammillary body atrophy.

## 2. Case Report

### 2.1. First Episode

A 16-year-old girl was admitted to the surgical ward for evaluation of recurrent vomiting of 3-week duration. Past medical history was noted for congenital multiple intestinal atresia and bilateral clubfeet. She had a prolonged hospitalization for multiple intestinal resections and she was on total parenteral nutrition for few months. She was followed by the pediatric gastrointestinal service and she was noticed to have failure to thrive. Her weight and height were below 3rd percentile throughout her childhood. She was on normal diet and was not compliant to her dietary supplements to increase her weight. She had normal cognitive function and attended ordinary schools. She had menarche in time. Her clubfeet, which was secondary to hereditary in nature according to the pediatrician's notes, did not interfere with her daily activities. According to the pediatrics' notes, her yearly follow-up revealed borderline iron deficiency anemia and low vitamin D levels. Her previous investigations revealed partial intestinal obstruction secondary to adhesions related to her previous intestinal surgeries.

Upon admission, she was kept nil by mouth and she was given intravenous fluids only. There was no history of confabulation or fluctuation in the level of consciousness. One week later, she developed acute diplopia, vertigo, and ataxia. Her general examination was unremarkable except for poor growth (height: 143 cm, weight: 34 kg; both were below the 5th percentile). On neurological examination, she was fully conscious oriented with normal speech and language. Cranial nerve examination revealed symmetric pupillary reaction to light and accommodation with normal fundoscopy. She had impaired bilateral horizontal gaze secondary to bilateral CN VI paresis. The rest of cranial nerve examination was unremarkable. Motor exam revealed normal tone and power but her deep tendon reflexes were diminished and planters were down-going. Sensory exam was unremarkable. Bilateral upper limb dysmetria was noted with normal gait.

Considering her prior history of poor nutrition and acute neurological presentation following recurrent vomiting, the possibility of vitamin deficiency was considered. She was given IV thiamine 200 mg once daily for a week along with injectable Vitamin B12 1000 ugm and oral folic acid 5 mg once daily. Laboratory investigations revealed Hb = 11.0/l, RBC = 3.66 × 10^12^/L (3.8–4.8), MCV = 90 (83–101), MCH = 30 pg (27–32), MCHC = 335 (315–345), and WBC = 8.4. Several vitamin levels were subsequently measured revealing the following results: B1 = 125 ng/mL (20–100), B2 = 231 ng/mL (75–300), B6 = 15.4 ng/mL (7–30), Vitamin C < 5.7 umol/L (> 11.4) and Vitamin D = 22 (30–75) which was measured 3 months prior to her admission. MRI brain was unremarkable. She started to improve in 48 hours. Diplopia completely resolved along with her eye movements within a week except for bilateral gaze-evoked-horizontal nystagmus. She was discharged without any neurological symptoms on oral thiamine 50 mg once a day.

### 2.2. Second Episode

**Figure 1:**
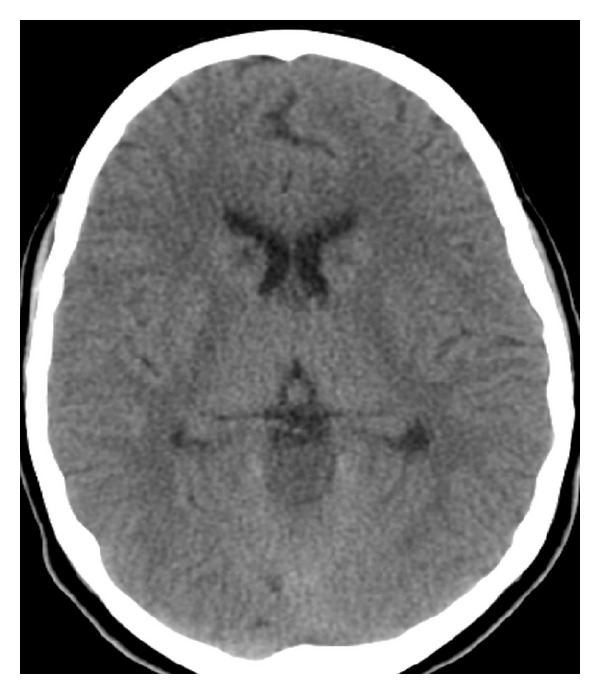
Axial images of Computed Tomography (CT) of brain showing bilateral hypodensities of the caudate nuclei.

**Figure 2:**
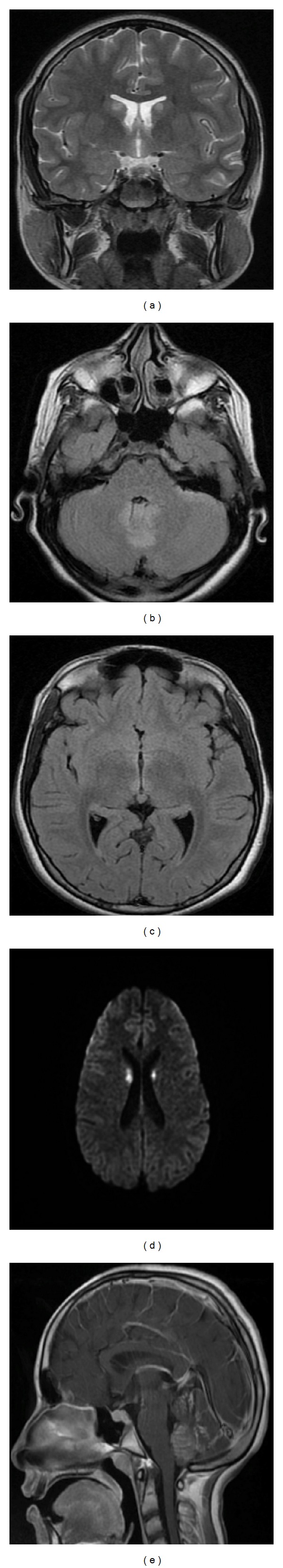
Magnetic Resonance Images performed during the second episode. (a) T2 coronal images showing hyperintense signals in caudate nuclei bilaterally. (b)-(c) Axial FLAIR images showing hyperintense signals in vermis and periaqueductal areas. (d) Axial DWI showing restricted diffusion in caudate nuclei. (e) Sagittal T1 with contrast images showing enhancement of the vermis.

**Figure 3:**
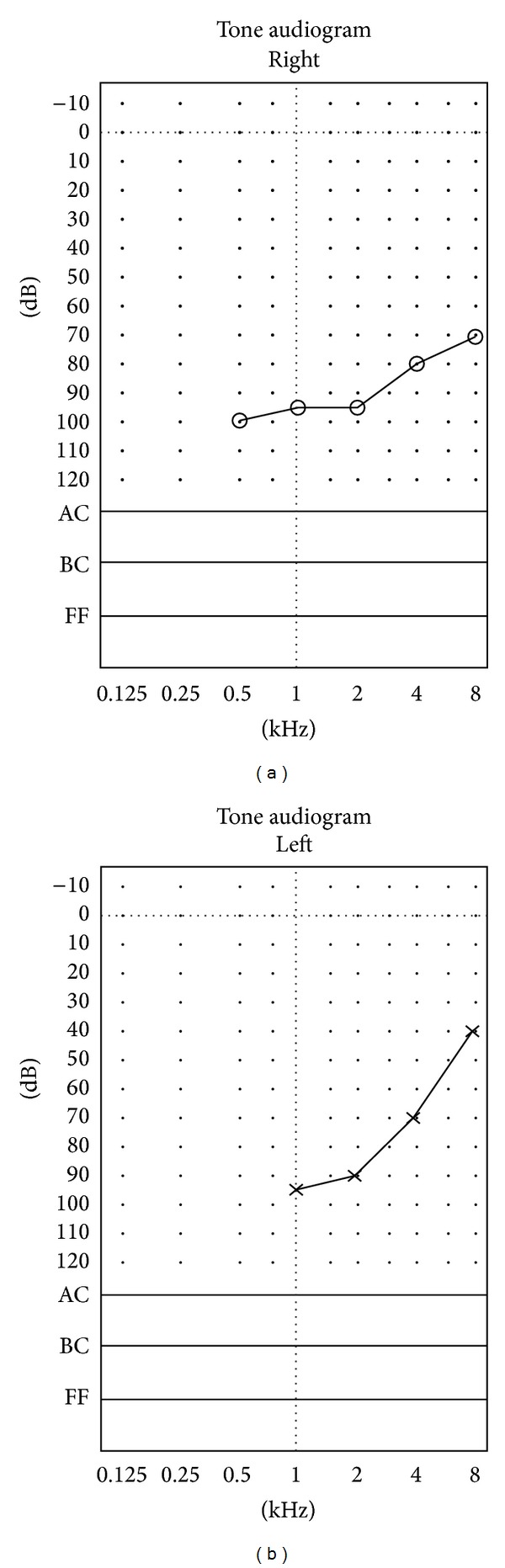
An audiogram revealed bilateral sensorineural deafness (performed during the second episode).

**Figure 4:**
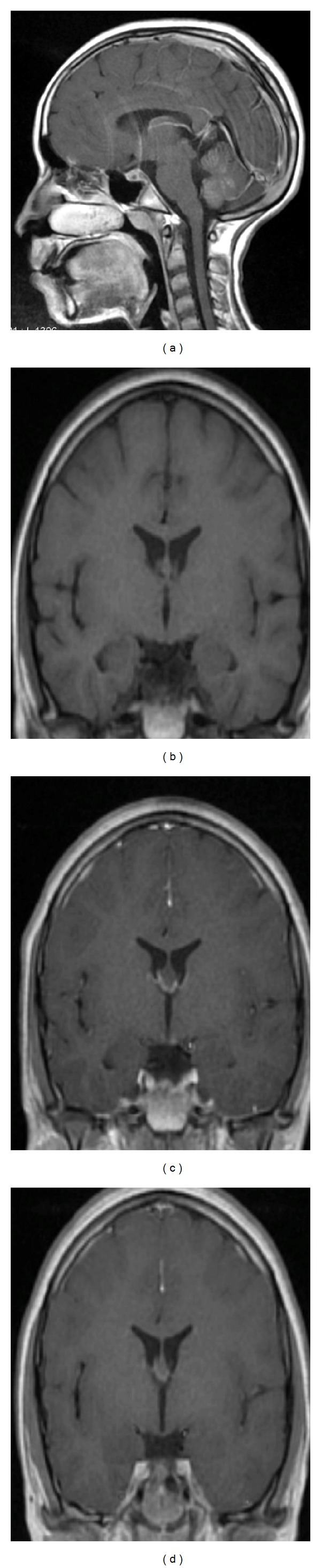
(a) Sagittal T1 with contrast image showed persistence of gadolinium in a follow-up study conducted 4 months later. (b)–(d) Sequential coronal T1 images showing the baseline mamillary body when MRI was performed during the first episode (b), enhancement of mamillary body during the second episode (c), and atrophy of mamillary body in a follow-up MRI performed 4 months later (d).

Four months later, she presented to the emergency department with 2-week history of severe headache, tinnitus, bilateral hearing impairment and diplopia, which were preceded by recurrent vomiting for 10 days. There was no history of confabulation or any change in the level of consciousness. She was mildly dehydrated, afebrile and had normal ear, nose and throat examinations. There were no meningeal irritations. On neurological examination, she was fully conscious, alert and oriented to time and place. Cranial nerve examination was noted for bilateral lateral rectus paresis (secondary to bilateral CN VI palsies) and bilateral severe sensorineural deafness. Her blood parameters revealed the following: hemoglobin 14.0, RBC 4.61, MCV 88, MCH 30, MCHC 347, WBC 8.2. Serum electrolytes and renal and liver function tests were within normal limits. Urgent noncontrast CT brain showed symmetrical bilateral caudate nucleus hypodensities (Figure 1). Due to her headache and bilateral subcortical lesions, the possibility of cortical venous thrombosis was considered and CT venography was ordered and it was negative. She was given IV thiamine 100 mg once daily for five days along with intensive hydration. Her headache disappeared by the next day and the tinnitus and diplopia improved within two days while deafness persisted. MRI brain revealed T2/FLAIR hyperintense signals of bilateral caudate nucleus, mamillary body, periaqueductal region and vermis (Figure 2). In addition, hyperintensity of caudate nuclei in Diffusion Weighted Images (DWI) and gadolinium enhancement of vermis and maxillary bodies were noted (Figure 2). Deafness persisted till her 4-month follow-up. Audiometry indicated severe bilateral hearing impairment (Figure 3). A follow-up MRI 4 months later, revealed partial resolution of vermian lesion, with atrophy of mamillary body (Figure 4).

## 3. Discussion

Thiamine deficiency in nonalcoholic patients can develop by four mechanisms (reduced availability, impaired utilization, accelerated usage and increased losses). Thiamine pyrophosphate is the active form of thiamine in CNS and is a cofactor for vital metabolic pathways such as pentose phosphate shunt, glycolysis and citric acid cycle. In glial cells and neurons, these metabolic pathways play a direct role in synthesis of myelin, nucleic acids, neurotransmitters and ATP [6]. In healthy person, body reserve of thiamine is sufficient for up to 18 days. If not supplemented, thiamine storage usually depletes in 2-3 weeks. Within 4 days of deficiency, cytotoxic edema in brain sets in, and after 7–10 days, vasogenic edema and breakdown of BBB occur due to endothelial cell dysfunction, nitrous oxide production and glutamate release. Irreversible neuronal necrosis occurs after 2 weeks due to breakdown of neuronal DNA and lactic acid production [7].

WE's classic triad consists of encephalopathy, ophthalmoplegia and ataxia. The presence of the classic triad is described in 53.9% alcoholics and 33.6% in nonalcoholics [3]. Our case lacked the presence of encephalopathy likely due to the quick response to rapid correction of dehydration and prompt institution of parental vitamin B1 supplementation. The diagnosis of WE, when presented with atypical features, might be missed due to lack of physician's awareness. WE-associated deafness is rare [8–10]. Jethava and Dasanu reported a 44-year-old female who had bilateral hearing impairment secondary to WE following bariatric surgery. There were symmetrical thalamic lesions on MRI which improved 8 weeks later [8]. Zhang et al. reported another case of reversible sensorineural hearing loss after 2-day of thiamine deficiency in a 23-years-old male who was suffering from acute pancreatitis. MRI showed hyperintense signals on diffusion in bilateral inferior colliculi, superior colliculi, postero-medial thalamus and inferior cerebellar peduncles. However, our patient did not show any improvement in her hearing loss after 4 months of follow-up. We were not sure if this would be permanent. In a long-term follow-up study of 11 infants, auditory neuropathy resolved in 5, remained permanent in 2, deteriorated in 1 over 6–8 years of follow-up [11].

Many clinic-pathological studies indicated that in approximately 80% cases, the diagnosis of WE could not be done during life [12, 13]. Similarly, substantial time delays had been reported since WE was misdiagnosed with a psychiatric disease [14]. The sensitivity of the classical triad was low. In an autopsy review study of alcoholic WE, 16% of patients had all 3 classical signs, 29% had 2 signs, 37% had 1 sign and 19% had no sign [14]. This indicated that classical clinical signs did not reflect the variations or atypical presentations. In 1997, Caine et al. proposed new operational criteria where they added a dimension for malnutrition [15]. By using these criteria in alcoholics, the diagnosis of WE could be made if 2 out of 4 signs (eye signs, cerebellar signs, mild memory impairment or confusion and signs of malnutrition) were present. These criteria increased the sensitivity and the specificity to 85% and 100% respectively in neuro-pathologically confirmed WE [15].

With respect to neuroimaging, the sensitivity of brain CT is low in acute presentation compared to MRI. Hypodensity along the 3rd ventricle was seen in only 13% of patients in a series of 35 cases [16]. Antunez et al. described hypodensity of fornices as an atypical case [17]. The important finding of mamillary body involvement due to cytotoxic edema presented in our case was not previously documented by CT studies. MRI is the investigation of choice due to its high sensitivity of 53% and specificity of 93% [16]. Typical involved locations were thalami, mamillary bodies, tectal plate and periaqueductal areas. Mamillary body enhancement and atrophy were more consistently reported with alcoholic WE. Atypical location of lesions was more common in nonalcoholic WE [17]. Atypical lesions could be located in cerebellum, vermis, cranial nerve nuclei (hypoglossal, medial vestibular, facial and abducent nuclei), red nucleus, dentate nucleus, caudate nuclei, splenium, and cerebral cortex [18–20]. Mamillary body atrophy was not seen in most of the nonalcoholic WE cases. Contrast enhancement of lesions was highly variable and mamillary body enhancement could be the only sign of WE [19–21]. Enhancement of mamillary bodies was noted in our case during the acute phase of the second attack [22]. Death was reported in a 24-year-old nonalcoholic female who developed WE [19]. Cortical involvement was associated with poor prognosis or coma [23].

It is postulated that heterogeneity of lesions could be due to disease severity, acuteness of the disease and timing of imaging [5, 23]. In our case, sequential MR studies showed normal appearance of maxillary body in first episode, an enhancement during 2nd episode and an atrophy during follow up. There were limited available data on DWI/ADC use in the literature. Restricted diffusion suggesting cytotoxic edema was noted by Rugilo et al. [24]. The role of DWI/ADC is not yet clear in detecting acute lesions. Few reports on MR spectroscopy suggested that lactate peak was indicative of an impaired local metabolism [24, 25]. Reversible restricted diffusion on DWI (high signal) and low signal on ADC could be seen in some structures suggesting cytotoxic edema. However, hypo-intense ADC in association with hyperintense DWI might be indicative of cytotoxic edema as described in pathological studies [25].

## 4. Conclusions

Our case underscores the importance of high index of suspicion of WE in high-risk subjects prone to nutritional deficiency. Recurrence may differ in presentations and can present with atypical features such as neurosensory hearing loss. Bilateral caudate nuclei hypodensities on CT and gadolinium-enhancement and atrophy of mamillary body on MRI can be seen in nonalcoholic WE.

## References

[1] Kornreich L, Bron-Harlev E, Hoffmann C (2005). Thiamine deficiency in infants: MR findings in the brain. *American Journal of Neuroradiology*.

[2] Isenberg-Grzeda E, Kunter HE, Nicholson SE (2012). Wenicke-Korsakoff-syndrome: under-recognized and under—treated. *Psychosomatics*.

[3] Galvin R, Bråthen G, Ivashynka A, Hillbom M, Tanasescu R, Leone MA (2010). EFNS guidelines for diagnosis, therapy and prevention of Wernicke encephalopathy. *European Journal of Neurology*.

[4] Tanasescu R (2009). Wernicke’s encephalopathy in general neurological practice: short considerations on the need for revision (I). *Romanian Journal of Neurology*.

[5] Stenerson M, Renaud D, Dufendach K (2013). Recurrent Wernicke encephalopathy in an adolescent female following laparoscopic gastric bypass surgery. *Clinical Pediatrics*.

[6] Manzo L, Locatelli C, Candura SM, Costa LG (1994). Nutrition and alcohol neurotoxicity. *NeuroToxicology*.

[7] Sechi G, Serra A (2007). Wernicke’s encephalopathy: new clinical settings and recent advances in diagnosis and management. *The Lancet Neurology*.

[8] Jethava A, Dasanu CA (2012). Acute Wernicke encephalopathy and sensorineural hearing loss complicating bariatric surgery. *Connecticut Medicine*.

[9] Zhang S-Q, Guan Y-T (2012). Acute bilateral deafness as the first symptom of wernicke encephalopathy. *American Journal of Neuroradiology*.

[10] Buscaglia J, Faris J (2005). Unsteady, unfocused, and unable to hear. *American Journal of Medicine*.

[11] Attias J, Raveh E, Aizer-Dannon A, Bloch-Mimouni A, Fattal-Valevski A (2012). Auditory system dysfunction due to infantile thiamine deficiency: long-term auditory sequelae. *Audiology and Neurotology*.

[12] Harper C (1983). The incidence of Wernicke’s encephalopathy in Australia. A neuropathological study of 131 cases. *Journal of Neurology Neurosurgery and Psychiatry*.

[13] Lindboe CF, Loberg EM (1989). Wernicke’s encephalopathy in non-alcoholics. An autopsy study. *Journal of the Neurological Sciences*.

[14] Harper CG, Giles M, Finlay-Jones R (1986). Clinical signs in the Wernicke-Korsakoff complex: a retrospective analysis of 131 cases diagnosed at necropsy. *Journal of Neurology Neurosurgery and Psychiatry*.

[15] Caine D, Halliday GM, Kril JJ, Harper CG (1997). Operational criteria for the classification of chronic alcoholics: identification of Wernicke’s encephalopathy. *Journal of Neurology Neurosurgery and Psychiatry*.

[16] Antunez E, Estruch R, Cardenal C, Nicolas JM, Fernandez-Sola J, Urbano-Marquez A (1998). Usefulness of CT and, MR imaging in the diagnosis of acute Wernicke’s encephalopathy. *American Journal of Roentgenology*.

[17] Swenson AJ, St. Louis EK (2006). Computed tomography findings in thiamine deficiency-induced coma. *Neurocritical Care*.

[18] Zuccoli G, Pipitone N (2009). Neuroimaging findings in acute Wernicke’s encephalopathy: review of the literature. *American Journal of Roentgenology*.

[19] Fei G-Q, Zhong C, Jin L (2008). Clinical characteristics and MR imaging features of nonalcoholic Wernicke encephalopathy. *American Journal of Neuroradiology*.

[20] Zuccoli G, Gallucci M, Capellades J (2007). Wernicke encephalopathy: MR findings at clinical presentation in twenty-six alcoholic and nonalcoholic patients. *American Journal of Neuroradiology*.

[21] Zuccoli G, Cruz DS, Bertolini M (2009). MR imaging findings in 56 patients with wernicke encephalopathy: nonalcoholics may differ from alcoholics. *American Journal of Neuroradiology*.

[22] Shogry MEC, Curnes JT (1994). Mamillary body enhancement on MR as the only sign of acute Wernicke encephalopathy. *American Journal of Neuroradiology*.

[23] Escobar A, González M, Aguayo G, García-Ramos GS (2009). Acute Wernicke’s encephalopathy. Comparison among the clinical features, magnetic resonance images and neuropathology findings. *Revista Mexicana de Neurociencia*.

[24] Rugilo CA, Roca MCU, Zurru MC, Capizzano AA, Pontello GA, Gatto EM (2003). Proton MR spectroscopy in Wernicke encephalopathy. *American Journal of Neuroradiology*.

[25] Halavaara J, Brander A, Lyytinen J, Setälä K, Kallela M (2003). Wernicke’s encephalopathy: is diffusion-weighted MRI useful?. *Neuroradiology*.

